# A methodology for predicting tissue-specific metabolic roles of receptors applied to subcutaneous adipose

**DOI:** 10.1038/s41598-020-73214-w

**Published:** 2020-11-11

**Authors:** Judith Somekh

**Affiliations:** Department of Information Systems, University of Haifa, Haifa, Israel

**Keywords:** Systems biology, Computational biology and bioinformatics, Data processing, Functional clustering, Machine learning

## Abstract

The human biological system uses ‘inter-organ’ communication to achieve a state of homeostasis. This communication occurs through the response of receptors, located on target organs, to the binding of secreted ligands from source organs. Albeit years of research, the roles these receptors play in tissues is only partially understood. This work presents a new methodology based on the enrichment analysis scores of co-expression networks fed into support vector machines (SVMs) and k-NN classifiers to predict the tissue-specific metabolic roles of receptors. The approach is primarily based on the detection of coordination patterns of receptors expression. These patterns and the enrichment analysis scores of their co-expression networks were used to analyse ~ 700 receptors and predict metabolic roles of receptors in subcutaneous adipose. To facilitate supervised learning, a list of known metabolic and non-metabolic receptors was constructed using a semi-supervised approach following literature-based verification. Our approach confirms that pathway enrichment scores are good signatures for correctly classifying the metabolic receptors in adipose. We also show that the k-NN method outperforms the SVM method in classifying metabolic receptors. Finally, we predict novel metabolic roles of receptors. These predictions can enhance biological understanding and the development of new receptor-targeting metabolic drugs.

## Introduction

The human system, as any other biological system, always aiming to achieve a state of homeostasis, responds to different conditions through activating feedback control loops between its sub-systems, organs and tissues. For example, to ensure whole organism survival, the endocrine system preserves long feedback loops of ligands secretion and receptors binding to maintain glucose or energetic balance. Ligand–receptor secretion and binding are accomplished by molecules, i.e., ligands, secreted into the blood stream from source organs that bind to receptors located on both the cell surface and within the cells of target organs. This complex network of whole-body ligand–receptor interactions serves as the information transducer of these feedback loops. Understanding these receptor roles is pivotal in the field of modern medicine. Receptor dysregulation underlies the etiology of many human diseases (e.g., diabetes^1^) and prescription drugs are designed to affect the regulation of receptors, e.g., by distrupting the interaction to the ligand, and produce therapeutic changes in the function of related biological systems^1,2^. Moreover, receptors serve as targets for virus invasion of cells, e.g., the ACE-2 receptor is responsible for the entrance of the COVID-19 virus into the lungs^3^. Albeit years of research, our present-day understanding of the tissue-specific functions of many receptors and their ligand intercellular signalling networks is still incomplete. Developing drugs continues to be a challenge, as advances in scientific knowledge of receptors has been relatively slow, being based on laborious experimentation that typically precedes testing one or two receptors at a time in one or two tissues.

The advent of ultrahigh-throughput sequencing technologies and algorithmic advancements now enable us to investigate systematically and simultaneously hundreds of genes coded to receptors. A recent computational work^4^ defined cross-tissue expression of ligand–receptor pairs by merely measuring the expression levels of ligands and receptors across 144 cell types. A common task of analysis of gene expression data is to detect gene–gene co-expression networks. These gene co-expression networks are based on the “guilt by association” concept that is related to the fact that functionally related genes are co-expressed^5^. Such networks are used to identify the functional roles of genes whose function is unknown by relating their co-expression networks to known biological processes. For example, Horan et al. annotated genes of known and unknown function by large-scale coexpression analysis^6^. The Weighted Gene Co-expression Network Analysis (WGCNA)^7^ is the most popular algorithm for specifying co-expression networks. The algorithm groups related genes into gene modules (clusters) based on their co-expression patterns and topological similarity to neighbour genes in the network. Machine learning approaches are gaining popularity for gene expression analysis^8,9^ and the support vector machines (SVMs) are one of the most widely used type of machine learning algorithm for solving binary classification problems^10^. SVMs have successfully classified functional modules and protein interaction networks from gene expression data^8,9^. The binary SVM classifier is based on defining a hyperplane that distinguishes between the positive labeled data (e.g., metabolic receptors) and the negative labeled data (e.g., non-metabolic receptors) based on the feature space, the properties of the data. The k-NN (k-nearest neighbours) algorithm is a distance-based approach that classifies the data points based on the known classification of their neighbours^11^.

The GTEx project^20^ includes a unique collection of thousands of samples of RNA-seq gene expression data across multiple tissues collected from hundreds of donors. Using this data and focusing on metabolic receptors and adipose tissue, we ask several questions: (1) Is expression of genes coded to receptors widely correlated within tissues? And in adipose in specific? (2) How can we use this data to infer the metabolic roles of receptors in tissues and to detect new metabolic receptors, not thought of as being members of a specific classically defined metabolic system? Together, answers to these questions can begin to delineate a comprehensive view of the metabolic network signalling.

**Figure 1:**
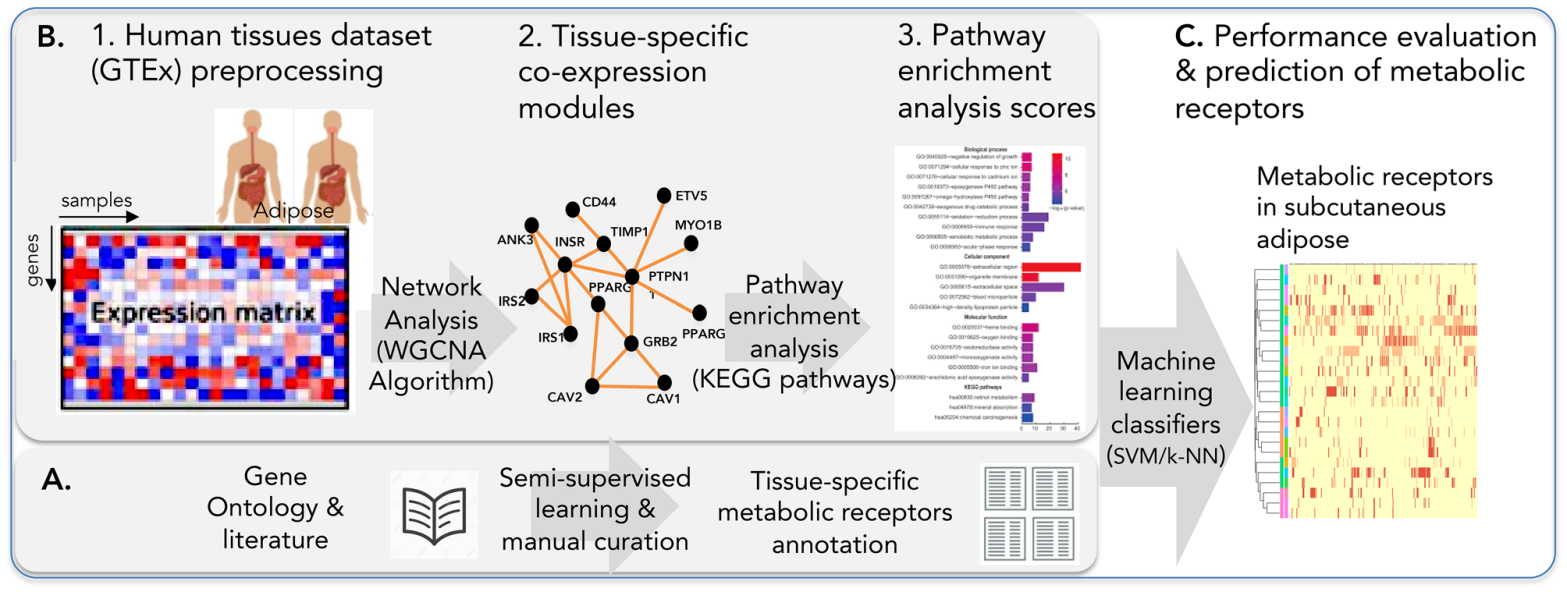
Schematic view of the new computational methodology.

Here we present a new computational methodology to predict tissue-specific receptor metabolic functionality, which we applied to subcutaneous adipose. The methodology incorporates three steps A, B and C (see Fig. 1) and is based on our new finding that metabolic receptors are co-expressed, among themselves and with other genes. In Step A an annotated list of metabolic and non-metabolic receptors in adipose was constructed using a semi-supervised approach and literature-based validation. In Step B we used the (WGCNA) algorithm^7^ for co-expression network analysis to generate gene modules (clusters) in subcutaneous adipose followed by their pathways enrichment analysis. We used the enrichment scores to train SVMs and k-nearest neighbour (k-NN) classifiers and compared their performance, in Step C. Finally, we used the classifiers to predict new metabolic receptors, having previously unknown metabolic functions, in adipose. We used an extensive list of ~ 700 receptors for the full analyses and predictions.

## Results

The new computational methodology predicts tissue-specific roles of metabolic receptors in subcutaneous adipose and comprises the following steps.

### Step A: Subcutaneous adipose receptor labeled list

Supervised learning requires an initial labeled list of known metabolic (positive examples) and non-metabolic (negative examples) receptors in a tissue for the training, performance evaluation and construction of the classifier.

We chose to study adipose tissue^13^ since it is a highly active endocrine and metabolically important organ, with the ability to modulate glucose homeostasis, energy expenditure, lipid metabolism, and peripheral inflammation. In addition, the existing knowledge about its metabolic receptors roles is extensive and, experimentally, it was robustly tested in comparison to other tissues.

One main challenge for us was to detect the receptors that exhibit metabolic roles in adipose and those that do not. We note that we use the term metabolic receptors to include receptors related to the metabolic/endocytosis/growth regulation system^14–16^. This knowledge is not easily available since public databases, such as KEGG, do not include a metabolic receptor classification in general or a tissue-specific metabolic receptors classification in particular. For example, the KEGG database includes the “Neuroactive ligand-receptor interaction” pathway that consists of a combination of metabolic and non-metabolic receptors. The insulin receptor is included in its own pathway, the KEGG insulin signalling pathway. In addition to the "pure" metabolic receptors a receptor may exhibit ubiquitous roles across the whole body, e.g., a known inflammation-related cytokine receptor which we possibly label as a non-metabolic negative example, may exhibit metabolic roles in adipose. An example is the cytokine receptor TNFRSF21, a tumor necrosis factor receptor superfamily member 21, that is include in the KEGG “Cytokine-cytokine receptor interaction” but is also related to the “regulation of lipid metabolic process” in GO (Gene Ontology)^17,18^.

To construct the initial positively labeled receptors list, we gathered a list of 33 metabolic receptors known from the literature to be related to the regulation of growth, endocytosis and metabolism^14–16^. The reader is directed to Supplemental Table S1 for this list and additional references for the metabolic regulation roles of these receptors. After filtering for the receptors not available in adipose (e.g., not included in any module in subcutaneous adipose; see the “Methods” section), we received a total of 17 receptors as our initial positive example set. To enrich the positive and negative examples set, we used the SVM PU (positive unlabeled) bagging algorithm^9,19^, that is suitable in using a limited number of known positive examples to successfully discriminate between the positive and negative examples in an unlabeled data set (see “Methods” section). This way, we added an additional 35 metabolic receptors as positive examples, which we verified manually as being metabolic using the GO (Gene Ontology)^17,18^ database and a thorough literature review (see “Methods” section and supplemental Table S2 for their list and references to experimental evidence).

As the negative examples two distinct groups were used: (1) cytokine receptors from the KEGG “Cytokine-cytokine receptor interaction” pathway that were not included in the positively labeled examples (total of 61 receptors) and (2) a total of 55 receptors inferred to be strongly negative (negative rate > 0.8 out of 100 experiments, see “Methods” section) by the PU bagging algorithm. Pathway enrichment analysis on the second group's receptors shows that they were significantly enriched (adjusted *p* value < 10^−16^) with 17 cytokine receptors from the “Cytokine–cytokine receptor interaction” and nine different neuroactive receptors from the KEGG “Neuroactive ligand–receptor interaction” pathway (see supplementary Table S3).

### Step B1: Data preparation

The GTEx subcutaneous adipose gene expression data was filtered, pre-processed and corrected for batch effects as described in the “Methods” section.

### Steps B2–3: Co-expression modules and pathway enrichment analysis

**Figure 2:**
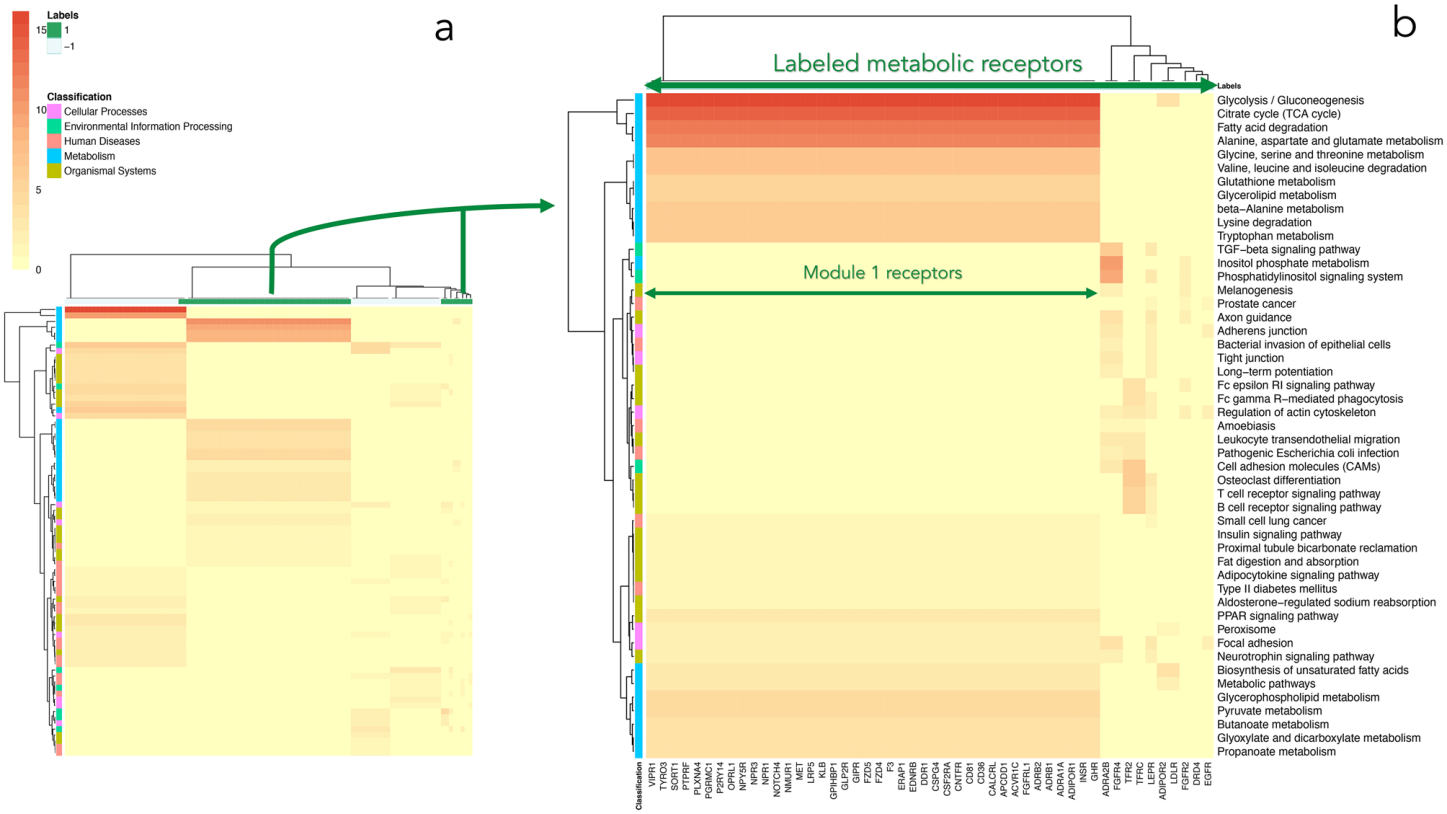
Pathway enrichment analysis of the labeled metabolic receptors related modules in subcutaneous adipose. A heatmap of log-transformed p-values (adjusted for multiple correction) of the KEGG pathways enrichment analysis is presented. (**a**) Enriched pathways for the metabolic and non-metabolic receptors used for training. It can be seen that the metabolic receptors (highlighted in green in the annotated columns) form a metabolic cluster (highlighted in the annotation rows to the right in turquois and corresponding to the KEGG metabolism hierarchical classification). (**b**). Focusing on the metabolic receptors related modules shows that they are highly enriched with various metabolic pathways. The rows represent the KEGG pathways, and the columns, the receptors [e.g., insulin receptor (INSR)]. Multiple metabolic receptors are included in Module 1 in subcutaneous adipose, which is enriched with metabolic pathways.

A total of 17 modules, co-expression networks, were generated by the WGCNA algorithm (see “Methods” section) for subcutaneous adipose tissue. Following modules construction, we conducted pathway enrichment analysis using KEGG pathways. A heatmap of the pathway enrichment scores of the metabolically annotated receptors in subcutaneous adipose (positive examples) and negative examples (group 2), which we used in our analysis, is presented in Fig. 2. The presented enrichment scores are the − log_10_ transformation of the adjusted *p* value enrichment scores (see “Methods” section). Rows and columns with zero enrichments were removed from the representation. Figure 2a shows that the positive metabolically annotated receptors (highlighted in the column’s annotation in green) form a strong cluster annotated by KEGG hierarchies to constitute the metabolic process (highlighted on the row’s annotation to the right in turquois) and to include various metabolic pathways. Figure 2b focuses on the positive metabolic examples to show their enriched metabolic pathways. These include, among others, “Glycolysis”, “Fatty acid degradation”, “Pyruvate metabolism” and “Glycine, serine and threonine metabolism”. Most metabolic receptors are co-expressed and included in Module 1 of subcutaneous adipose (the left cluster in Fig. 2b), e.g., insulin receptor (INSR), adiponectin receptor 1 (ADIPOR1), and growth hormone receptor (GHR). Module 1 receptors network is presented in supplemental Figure S1 to highlight the receptors’ connectivity and correlation with the module eigengene.

### Step C: Validation and prediction.

#### C.1. Classifier construction and validation

We used supervised approaches, linear SVM and k-NN, to solve the problem of binary classification of receptors to be “metabolic” and “non-metabolic” in subcutaneous adipose tissue (further elaboration will be found in the “Methods” section). We used tenfold cross-validation to evaluate the performance of our classifiers. We used each of the two negative groups (cytokines and the inferred group) separately. The performance of the classifiers for the positive labels and each of the negative labels is presented in Table 1. Columns 4–7 are the false positive (FP), false negative (FN), true positive (TP), and true negative (TN). Columns 8–11 are the sensitivity, specificity and accuracy measure of overall performance and the MCC (see “Methods” section). The accuracy of all calculations is > 0.9. k-NN outperforms SVM with an accuracy of 0.98 and a Matthews Correlation Coefficient (MCC) measure of 0.96 for the inferred negative group. k-NN classified 50 positive examples (TP) correctly as opposed to 44 for the SVM linear classifier. For the cytokines receptors as the negative group, the classifiers produced similar results with 42 TPs. There were few FPs (≤ 1), which means that our approach will not label a non-metabolic receptor as a metabolic one. We repeated the experiment with SVM and k-NN ten more times with different random splits of the data. The variance introduced by the random splitting of the data was very small (< 10^−5^) relative to the mean.

**Table 1:** Comparison of performance evaluation of linear SVM and k-NN classifiers (using the Euclidian distance) for metabolic receptors classification in subcutaneous adipose tissue. The labeled examples include 52 positive examples and 55 negative examples (rows 1 and 2) and 61 cytokines receptors as negative examples (row 3).

	Method	Negative group	TP	TN	FP	FN	Sensitivity	Specificity	Accuracy	MCC	FP receptors	FN receptors
1	k-NN	Inferred receptors	50	55	0	2	0.96	0.96	0.98	0.96	NA	TFRC
												TFR2
2	SVM	Inferred receptors	44	55	0	8	0.85	0.87	0.93	0.86	NA	ADIPOR2
												DRD4
												EGFR
												FGFR2
												LDLR
												LEPR
												TFR2
												TFRC
3	SVM/k-NN	Cytokines receptors	42	60	1	10	0.81	0.86	0.9	0.81	TNFRSF21	ADIPOR2
												ADRA2B
												DRD4
												EGFR
												FGFR2
												FGFR4
												LDLR
												LEPR
												TFR2
												TFRC

TNFRSF21 receptor (Tumor necrosis factor receptor superfamily member 21) is a cytokine receptor that is misclassified as a non-metabolic receptor by both k-NN and SVM. TNFRSF21 is related to the “regulation of lipid metabolic process” in GO^17,18^ and predicted by our analysis to exhibit a metabolic role in adipose. When excluding TNFRSF21, no FPs are detected. TFR2 and TFRC are both misclassified as metabolic receptors by both classifiers.

#### C.2 Metabolic role predictions for unlabeled receptors

In addition to validating the classification accuracy of the classifiers using the labeled receptor list, we used the classifiers to classify unlabeled receptors. We trained linear SVM and k-NN models on all the labeled data (for the two negative groups separately resulting in 4 classifiers) and then used the four models to predict the unlabeled receptors. A total of 387 receptors were included in the analysis, i.e., were incorporated in a co-expressed module in subcutaneous adipose. Of these, 21 receptors were predicted by all the four classifiers to exhibit metabolic roles in subcutaneous adipose. These predictions may merit experimental testing. Table 2 presents the predicted metabolic receptors. We further discuss the supporting literature of their metabolic roles in the discussion section.

**Table 2:** Predicted metabolic roles of unlabeled receptors by four classification models in subcutaneous adipose.

	Predicted metabolic receptor
1	CD151
2	CD46
3	CD63
4	FZD9
5	GPR56
6	IL27RA
7	ITGA2B
8	ITGA7
9	ITGAE
10	ITGB1
11	LPHN1
12	P2RY12
13	PLGRKT
14	PLXNA2
15	PTH1R
16	RHBDL2
17	RTN4RL1
18	SCN4A
19	SDC1
20	SLC16A2/MCT8
21	TACR2

## Discussion

We present a new methodology to predict tissue-specific metabolic roles of receptors. We used linear SVM and k-NN classifiers on a feature space of pathway enrichment analysis scores of receptor-co-expressed modules. We applied our method on subcutaneous adipose expression RNA-seq data derived from the GTEx project^20^. As an initial required step, we combined semi-supervised learning and a manual literature review to construct a knowledge base of receptors that exhibit metabolic roles in subcutaneous adipose. We evaluated the performance of the classifiers (accuracy ≥ 0.9) to show that metabolic receptors can be recognized successfully using this new feature space. The k-NN method provides superior performance when compared to the linear SVM method, using our data. Additionally, we predict 21 new metabolic roles for receptors in adipose when analysing hundreds of unlabeled receptors.

Our approach is based on our recognition that known metabolic receptors are co-expressed. Although studies show that gene expression is affected by metabolic stimuli such as glucose intake e.g., the work of Vaulont et al.^21^ showed of gene transcription regulation by glucose, the mechanism that explains the co-regulation of metabolic receptors and gene expression in general is still poorly understood. A recent breakthrough study^22^ which discovered that insulin receptor stimulation drives genome-wide expression of metabolic genes, may explain part of this mechanistic rationale of the metabolic co-expression of receptors and genes. The study’s authors^22^ showed that upon binding of the insulin receptor to insulin, it is translocated into the nucleus and associates with a promoter in a genome-wide manner to regulate gene expression of multiple metabolic and insulin signalling pathway-related genes.

Machine learning approaches were used on gene expression data (the feature space) for classification of functional classes of genes and to infer protein interaction networks^8,9^. Our approach uses further computation on gene expression data to construct a higher-level feature space and classify the metabolic roles of receptors. Our enrichment scores that rate the gene’s co-expression network go beyond relating to a single gene, which makes our system more robust to gene–gene network perturbations and noise, and also reduces the number of features, from thousands of features (genes), into several hundreds of features (pathways), which may decrease overfitting and improves the classification accuracy.

Co-expressed module 1 in subcutaneous adipose is a metabolic module, enriched with multiple metabolic pathways (see Fig. 2) and includes 42 of the labeled metabolic receptors. One can say that metabolic receptors can be detected in an unsupervised manner, just by intuitively extracting the receptors from the metabolically annotated modules, e.g., module 1. So it is reasonable that the classifiers classify correctly these 42 receptors that are included in module 1. An additional 10 labeled metabolic receptors are included in separate modules (see Fig. 2b). Both classifiers classify correctly (rows 1–2 in Table 1) two additional receptors, ADRA2B and FGFR4, which are included in other modules. Our approach detects as metabolic these two additional receptors derived from other modules, which is less than intuitive to detect. Moreover, the k-NN classifier detects 8 out of these 10 as being metabolic. Our approach is highly accurate in detecting the negative examples as well. When excluding the misclassified negative example, the TNFRSF21 cytokine receptor (which may be metabolic in adipose), no FPs are detected.

Our network-based approach is generalizable and can be used on other tissues. The main obstacle is the unlabeled data in general and per each tissue specifically. For subcutaneous adipose we used a thorough literature review directed by a semi-supervised approach, PU SVM bagging, based on multiple classifiers starting from small initial positively annotated examples. The semi-supervised learning defined the negative labels and extended the positive labels, which we further verified manually using the literature. We also showed independently that our approach successfully classifies metabolic receptors against KEGG cytokine receptors list used as negative examples.

A powerful feature of our method is its ability to generate detailed testable hypotheses concerning the metabolic roles of specific receptors in specific tissue, i.e., adipose. For example, NPR1 and NPR3, reciprocal regulation of natriuretic peptide receptors, are predicted to be metabolic in adipose by our approach and got a positive rate of 98% out of 100 tests using the PU bagging algorithm while NPR-2 is predicted to be non-metabolic in adipose. Interestingly, NPR1 and NPR3 expression was found to be induced by insulin in adipose cells as opposed to NPR-2 whose expression levels were not changed^23^. Nevertheless, we note that our method is based on mRNA levels and may miss some of the metabolic receptors that are regulated at the protein level.

Our approach predicts a list of 21 receptors to regulate metabolic response in adipose. We predict that PLGRKT, a novel plasminogen (PLG) receptor whose roles in humans are poorly understood, is a metabolic regulator in subcutaneous adipose. PLGRKT^24–26^ (named Plg-RKT in mouse) is a plasmin receptor that is highly conserved across mammalian species and broadly expressed in human tissues^27^. PLGRKT is theorized to be part of a local catecholaminergic cell plasminogen activation system that regulates neuroendocrine prohormone processing^28^. It significantly enhances the conversion of its ligand, plasminogen into plasmin, by supporting binding of plasminogen activators that have a role in macrophage recruitment during inflammatory response^24^. Plasmin^26^ the ligand, exhibits a broad-spectrum proteolysis activity with cell surfaces that promotes cell migration during inflammation, wound healing and muscle regeneration. Plasmin has several receptors and the interplay between plasminogen and its receptors is known to regulate inflammation. Plasminogen activator inhibitor-1 (PAI-1) inhibits the generation of the key enzyme, plasmin, by inactivating both the tissue-type plasminogen activator (tPA) and the urokinase-type plasminogen activator (uPA)^29^. Interestingly PAI-1 production by adipose tissue is increased in obesity, and its circulating levels are high in type 2 diabetes^29,30^. Mice lacking PAI-1 were completely prevented from developing obesity and insulin resistance in comparison to WT mice on an HF diet^30^. Our results predict that PLGRKT is a regulator of cell metabolism in adipose. Just recently the metabolic roles of PLGRKT are started to be elucidated. The Plg-RKT receptor was shown to regulate the uptake of Lipoprotein(a), Lp(a), by liver cells^31,32^ and Plg-RKT deficiency significantly affected the growth rates of female mice^33^. In genome-wide association studies (GWAS), genetic susceptibility in PLGRKT was found to be related to obesity in 815 children^34^ and in 1965 cohorts^35^ as related to metabolic traits. Recent finding by Milles et al.^36^ support our prediction and propose that Plg-RKT regulates metabolic homeostasis in a mouse model and promotes healthy adipose function. They showed that Plg-RKT−/− mice gained significantly more weight, their total fat mass was significantly greater, and insulin signalling in their adipose tissue was significantly (80%) lower. Another receptor we predict to be metabolic is CD63, a cell surface receptor for TIMP1, which was shown to be highly differentially expressed between obese high fat diet mice and low fat diet mice in macrophage cells (CD9 cells) derived from adipose tissue^37^. An additional predicted receptor CD46 co-stimulates optimal human CD8+ T cell effector function via fatty acid metabolism^38^. Finally the GPR56 predicted metabolic receptor has a mechanistic link to pancreatic cells function^39^. We note that our method may detect pure, classically known, metabolic receptor but also non-pure metabolic receptors that may mediate metabolic-inflammatory responses in adipose, e.g., the predicted PLGRKT receptor that exhibits inflammatory and metabolic roles is conjectured to mediate metabolic-inflammatory responses in adipose.

In summary, our methodology established the first step in using gene expression data to predict the roles of receptors in tissues. In future work we plan to extend this work to multiple tissues. This understanding of receptor roles in tissues would be tremendously significant in many areas of systems biology, drug discovery and modern medicine.

## Methods

### Data pre-processing

GTEx RNA-Seq data of 53 human tissues and 8555 RNA-seq samples from 544 donors was downloaded from the GTEx database (^40^, v6), and their reads per kilobase per million (RPKM) values were log2-transformed. 19,814 protein-coding genes were retained. Outlier samples were filtered and all genes within each tissue were quantile normalized (to remove background and sample effects). Outliers removal included standardizing sample distances and flagging as outliers the samples with high negative standardized distance (SD <  −3), meaning more than three standard deviations from the mean. Genes with zero variance or missing samples were excluded from the calculation (e.g., for Adipose–Subcutaneous 262 genes were excluded). Genes having at least 0.1 RPKM in 80% or more of the samples were retained.

### Confounding factors adjustment

The type of death classification of the samples (DTHHRDY = death circumstances) is based on a four-point Hardy Scale. The ventilator death group samples were analysed since it had significantly shorter ischemic times, which preserve sample quality^41^. Our previous work^41^ showed that using some common methods for adjusting the heterogenous GTEx expression data for hidden confounding factors (e.g., using principle components) filters out many of the biological signals—which is relevant here. Thus *ComBat*^42^ from the R/Bioconductor package *sva*^42^ was used to adjust for known confounding factors, which has been shown to outperform other methods^41^. *ComBat* was applied to adjust for experimental batch, ischemic time (time that elapsed between actual death and sample extraction), gender and age. Due to the discrete nature of *ComBat*, the continuous ischemic time values were discretized into five bins, labeled 1–5, by partitioning them into 300 min intervals. Age includes the 20–80 year range and is partitioned into 10 year intervals (embedded in the GTEx dataset). Genes with zero variance per each batch group and type were removed. Batches with one sample within a batch were removed. Each batch was adjusted iteratively, accounting for the yet unadjusted batches in each iteration. *ComBat* successfully corrected the Adipose Subcutaneous gene expression profiles.

### Ligand–receptor pair list

A list of 692 known receptors was imported from an external referenc ^4^ which established a most comprehensive collection of ligands-receptors pairs by merging (1) multiple dedicated databases, the Ligand − Receptor Partners (DLRP)^43^, IUPHAR^44^ and Human Plasma Membrane Receptome (HPMR)^45^ databases, another (2) 2117 experimentally supported interactions in the HPRD^46^ and STRING^47^ databases, which included 1288 ligand–receptor pairs absent from (1) that was manually curated from the literature^4^. They finally curated a set of 2422 Ligand−Receptor interactions, of 692 distinct receptors.

### Co-expression module detection

The Weighted Gene Co-Expression Network Analysis (WGCNA) algorithm and relevant R package^7^ were used to identify co-expression networks. The algorithm calculated a similarity co-expression matrix using correlation *cor*(*i*,*j*) for all genes (the biweight midcorrelation measure that accounts for outliers, by assigning larger weights to values closer to medians, was used). The co-expression matrix is transformed into an adjacency matrix by using the soft thresholding power beta *β*, to which co-expression similarity is raised.

1 $$a_{ij} = \left( {0.5*\left( {1 + cor\left( {i,j} \right)} \right)} \right)^{\beta }$$

where *a*_*ij*_ represents the resulting adjacency that measures the connection strengths.

The power *β* = 12 was defined based on the criterion of approximating the scale-free topology of the network, as recommended in the original publication^7^. Then, a topological overlap matrix (TOM)^7^ was computed and converted into a dissimilarity TOM. The TOM calculated the topological similarity between every two neighbours in the network, i.e., evaluated the similarity of the neighbours for every two nodes. Finally, hierarchical clustering was used to produce a tree (dendrogram) from the dissimilarity TOM. By using dynamic tree cutting, different numbers of clusters (modules) were obtained from the tree. The resulting modules contained genes that are densely interconnected, to construct co-expression networks, names also modules, per each tissue. To define, in each module, the positively or negatively correlated genes the “signed” networks were used— meaning that the co-expressed modules include positive correlations between the nodes. Eigengenes are defined as the first principal component of the expression matrix for each module and represent the weighted average of the expression profile for each module. The eigengenes can be used to merge clusters and to screen for suitable gene targets by calculating module membership (*kME*) measures, also known as eigengene-based connectivity. This way the key driver genes were detected in each module.

### KEGG enrichment analysis of modules

The R package ‘clusterProfiler’^48^ generated enrichment analysis of the modules using KEGG pathways. All 294 KEGG pathways were used in the analysis and filtered for significant pathways with adjusted *p *values < 0.05 (adjusted for multiple corrections using the BH (Benjamini and Hochberg) method^49^).

## Support vector machines (SVMs)

Binary classification is the process of labeling the members of a given data set to be included in one of two groups on the basis of whether they have some set of similar properties or not. Two sets of examples, one set from each group, usually named as positive and negative examples, should be defined to train a binary classifier. SVM is a binary classification approach that was shown to perform well in a verity of settings^10^. A linear SVM constructs a hyperplane that separates the positive examples and the negative examples, based on their properties, of belonging to some class. Linear SVM is suited for a small number of samples to avoid overfitting^10^. We used the R package e1071 for the SVM computation^50^.

### Positive unlabeled (PU) SVM bagging

As supervised learning requires the definition of positive and negative examples for training. In most of the domains acquiring negative examples is more costly than the positive ones and sometimes even not possible. An example where we do not know whether it is positive or negative are called unlabeled examples. For example, if a receptor was shown experimentally to be metabolic, we label/annotate it as a positive metabolic, but we are uncertain and do not have the full knowledge to annotate the non-metabolic receptors, which are the unlabeled receptors. A set of methods called Positive Unlabeled (PU) learning algorithms^9^ are designed to achieve the task of learning from a limited set of positive examples and a large set of unlabeled examples, i.e., in the absence of negative examples. Most such methods use classical supervised classification methods such as a support vector machine (SVM) classifier. For example Kiliç and Tan^9^, compared eight PU learning algorithms to successfully reveal protein–protein interaction (PPI) networks from gene expression data using only positive prior knowledge of known protein–protein interactions. A successful algorithm for this aim is the PU bagging SVM algorithm^19^ where a random subsets from the unlabeled set is created and defined as the negative examples, and a classifier is trained from each of the subsets and the known positive examples. Finally, these multiple classifiers are merged to generate a negative and positive rate for each example. More specifically, the algorithm (1) creates a training set by combining all positive data points with a random sample from the unlabeled points, with replacement, (2) builds a classifier from this “bootstrap” sample, treating positive and unlabeled data points as positives and negatives, respectively, (3) apply the SVM classifier for prediction to whatever unlabeled data points were not included in the trained random sample – hereafter called OOB (“out of bag”) points – and record their scores, (4) repeat the three steps above many times and finally assign to each point the average of the OOB scores it has received, i.e., the rate of classifying negative/positive from all predictions of the gene. Only little improvement was seen in simulated and real data above 100 iterations of the algorithm^9,19^. The bagging SVM algorithm outperforms the state-of-the-art methods for PU learning^9,19^ and successfully discriminate between unlabeled positive and negative examples even when the number of known positives is limited. Our initial data consists of a limited positive set of known literature-reviewed metabolic receptors in adipose and unlabeled receptors set and we used the PU SVM bagging algorithm to extend the positive examples (which we verified to be positive) and define the negative examples.

### k-nearest neighbours algorithm (k-NN)

The k-nearest neighbours algorithm (k-NN) is a distance-based learning used for classification^11,51^, and is appropriate for binary classification of two classes. The algorithm uses as input the k closest labeled examples in the feature space. The most common distance measure is the Euclidean distance. A data point is classified by a plurality vote of its neighbours, with the data point being assigned to the class most common among its k nearest neighbours (k is a positive integer, typically small). The performance of k-NNs is very sensitive to the choice of k and an optimal k can be selected by various heuristic techniques^52^. A common way of choosing the empirically optimal k is by testing the error rate under a set of possible k values.

### Cross validation

Cross-validation^53^ is a method to evaluate the performance of a prediction model on data points that are not used to train the model. A popular method of cross-validations is sub-sampling (k-fold cross-validation). In k-fold cross–validation, as the name suggests, the dataset is randomly divided into k number of non-overlapping sets. During each iteration, one set is used as a test dataset and the rest are used for training the model. The test dataset is predicted by the trained model. This iteration is repeated k times, each time with a different train and test groups, and generates k different classification models. The performance statistics are calculated by summing in each distinct test group the true positives, true negatives, false positives and false negatives.

### Performance evaluation

We used sensitivity, specificity, accuracy and MCC (Matthews Correlation Coefficient)^54,55^ to evaluate the performance of the cross-validation analysis. The mathematical equations to calculate these parameters are as follows:

$$\begin{aligned} sensitivity & = \frac{TP}{{TP + FP}} \\ Specificty & = \frac{TN}{{TN + FN}} \\ Accuracy & = \frac{TP + TN}{{TP + FP + TN + FN}} \\ MCC & = \frac{{\left( {TP*TN} \right) - \left( {FP*FN} \right)}}{{\sqrt {\left( {TP + FP} \right)\left( {TP + FN} \right)\left( {TN + FP} \right)\left( {TN + FN} \right)} }} \\ \end{aligned}$$

where TP, TN, FP, FN and MCC represents true positive, true negative, false positive, false negative and MCC, respectively. Sensitivity and specificity correspond to the proportion of correct predictions of positive and negative examples. The overall correctly predicted examples were calculated by using accuracy, which was the arithmetic mean of sensitivity and specificity. The MCC is used as a measure of the quality of binary (two-class) classifications and is suitable for imbalanced datasets^54,55^. The MCC evaluates the balance between specificity and sensitivity^54,55^. The MCC value is equivalent to the Pearson’s phi correlation coefficient to represent the correlation coefficient between the trained and predicted values of binary classifications and lies between − 1 and 1. A highly successful predictor will have MCC value near to 1, while opposite and random predictions have MCC value − 1 and 0, respectively.

### Experimental design

We used linear SVM and k-NN classifiers on the features space to classify receptors to be “metabolic” and “non-metabolic” in subcutaneous adipose. The features space included 295 KEGG pathway enrichment analysis scores per each receptor, i.e., per each module that includes the receptor.

To construct the receptors labeled list we used a directing semi-supervised approach, PU SVM bagging to enrich our initial positive examples and generate the negative ones. We started with an initial data set of 17 positive examples (see supplemental Table S1) of known literature-based metabolic receptors in adipose^14–16^. We used the PU SVM bagging method, with number of iterations t = 100 and number of unlabeled samples k in each iteration as the number of the positive labeled examples k = 17, as suggested in^9^. We manually verified each receptor (with predicted positive rate > 0.7 gathered from the 100 iterations/classifiers) to be involved in metabolic/growth regulation (see supplemental Table S2 with related references). More specifically a receptor was annotated as metabolic if (1) the receptor or its ligand is related to metabolic/growth regulation GO functions or/and process^17,18^ or (2) an independent experimental evidence validates the receptor to regulate insulin/glucose/metabolism/growth (see supplemental Table S2 for all relevant references). This way we added 35 verified metabolic receptors to the initial 17 metabolic receptors. To evaluate the performance of our approach we used two groups as the negative examples, (1) cytokine receptors derived from the independent KEGG pathway "cytokine-cytokine receptor interaction", and (2) negative examples generated by the PU SVM bagging algorithm. We evaluated the pathway enrichment of the 55 strongest negative receptors (rate > 0.8) using the Enrichr web tool^56^ of group 2 (see supplemental Table S3).

For SVM classification, we used the optimized "cost" measure to yield the smallest error rate, using the tune() function. We used the R package e1071 for the SVM computation^50^.

For k-NN classifier we executed the knn() function from the R “class” library^57^ using Euclidian distance. The optimal k value (the number of nearest neighbours) was chosen by evaluating the error rate under each k = 1,2,...,10 and setting the maximal k with the lowest error rate.

The performance of the classifiers was tested by using a tenfold cross-validation. The labeled receptor list was randomly divided into 10 groups. Classifiers were trained by using 9/10 of the data and were tested on the remaining 1/10. This procedure was then repeated 9 more times, each time using a different 1/10 of the genes as a test group. The performance of the classifier was measured by examining how well the classifier identified the positive and negative examples in the test sets. Each receptor in the test set can be categorized as true positive that is a metabolic receptor in the tested tissue; true negative as a non-metabolic; A false positive that is classified by the classifier as a metabolic receptor but is a non-metabolic; false negatives receptor is placed as being non-metabolic by the classifier but is a metabolic receptor. We reported the performance measures and the number of receptors in each of these categories for the learning methods we tested and for both negative groups.

### Metabolic receptors prediction

The classification of metabolic function of unknown receptors was performed by first training the classifiers on the labeled receptors. The unlabeled receptors were then classified 4 times, each using SVM or k-NN with the two possible negative groups described earlier. The receptors that were predicted to be metabolic was classified as positive by each of the 4 models.

### Visualization tool

Module visualization (see supplemental figure S1) was performed using the Cytoscape tool^58^, which allows visualization and analysis of networks of biological associations and interactions.

## Supplementary information

Supplementary information.

## Data Availability

The GTEx data is available for download from (https://www.gtexportal.org/home/datasets).
