# Supplementary material for: A methodology for predicting tissue-specific metabolic roles of receptors applied to subcutaneous adipose
Source: Sci Rep. 2020 Nov 11;10:19535. doi: 10.1038/s41598-020-73214-w (PMC7659321; doi:10.1038/s41598-020-73214-w)
Supplement: Supplementary file 1 — Supplementary information. [file 41598_2020_73214_MOESM1_ESM.pdf]

Supplemental file:

A methodology for predicting tissue-specific metabolic roles of receptors applied to subcutaneous adipose

Judith Somekh[a]

[a] Department of Information Systems, University of Haifa, Haifa, Israel

Table S1. List of known hormones and their receptors, derived from [1,2,3]. "Relevant" columns mean that the receptor is present in the GTEx database or in the tested receptors list. Total of 17 metabolic receptors are valid (expressed or included in modules of subcutaneous adipose) to be tested in subcutaneous adipose.

	Receptor (gene symbol)	Hormone/Ligand	Relevant	Additional support of metabolic roles in adipose
1	**INSR**	Insulin (regulates glucose and metabolism [4, 5, 6])	+	[4, 5, 6]
2	**GHR**	Growth hormone(regulates growth and metabolism [7,8])	+	[7,8]
3	**ADIPOR1**	Adiponectin (regulates carbohydrate and lipid metabolism [9, 10])	+	[9, 10]
4	**ADIPOR2**		+	[9, 10]
5	**LEPR**	Leptin	+	[11]
6	GHSR	Ghrelin	NA (no module in Adipose)	
7	PPARG	peroxisome proliferator–activator receptor-γ (PPAR-γ)	NA (not in receptors list)	
8	GHRH receptor	GHRH	NA	
9	**TFRC** (alias TFR1)	Transferrin (regulates iron metabolism)	+	[12,13]
10	**TFR2**		+	[14, 15]
11	**LDLR**	LDL (Low-Density Lipoprotein)	+	[16]
12	DRD1	Dopamine	NA (no module in adipose)	
13	DRD2		NA (no modules)	
14	DRD3		NA (no modules)	
15	**DRD4**	Dopamine	+	[17]
16	PRLR	Prolactin	NA (no module in adipose)	
17	RYR1/2/3	Ryanodine	NA	
18	AVPR1A	Vasopressin	NA (not in a module in adipose)	
19	AVPR1B (AVPR3)		NA	
20	AVPR2		NA (not in a module in adipose)	
21	**ADRA1A**	Adrenergic hormone	+	
22	ADRA2A		NA (not in a module in adipose)	[18,19]

	Receptor (gene symbol)	Hormone/Ligand	Relevant	Additional support of metabolic roles in adipose
23	**ADRA2B**		+	[18,20]
24	**ADRB2** (b2-adrenergic receptor)	β-adrenergic receptor	+	[21]
25	**ADRB1**		+	
26	ADRB3		NA	
27	**EGFR**	epidermal growth factor (EGF)	+	
28	FGFR1	FGF21<br><br>Fibroblast growth factor 21	NA (not in a module in adipose)	
29	**FGFR2**		+	
30	FGFR3		NA (no module in adipose)	
31	**FGFR4**		+	
32	**FGFRL1**		+	
33	FGFR6		NA (no modules)	

Table S2. Positive receptors inferred by bagging and their experimental validation. We used GO processes to extract the involvement of the receptor in metabolic processes and literature review of the receptors and their ligands. SVM bagging results in 47 receptors with positive rate >0.98. We verified 33 of them to be metabolic. In addition, ADORA1, S1PR4, ERAP1 (positive rate > 0.7) were verified to be metabolic in adipose and were added to the positive examples. A total of 35 additional receptors were added to the 17 initial metabolic receptors (presented in supplemental table S1).

LRP5	NPR1	PGRMC1	PLXNA2	RTN4RL1	SLC16A2	NPY5R	CALCRL	OPRL1	PTH1R
CD36	DDR1	IL27RA	LPHN1	ACVR1C	CD46	CNTFR	ERAP1	F3	GIPR
GPR56	ITGA2B	NPR3	PTPRF	SCN4A	SORT1	VIPR1	CD151	CD81	CSF2RA
EDNRB	FZD5	ITGB1	KLB	MET	NMUR1	NOTCH4	TNFRSF21	CSPG4	FZD4
GLP2R	GPIHBP1	ITGA7	P2RY14	PLXNA4	TYRO3	APCDD1			

	Receptor	Evidence for metabolic roles in adipose and other tissues
1	ACVR1C/ALK7	GO processes: response to glucose, response to insulin, cellular response to growth factor stimulus, lipid storage, growth factor binding, cellular response to growth factor stimulus, negative regulation of insulin secretion, response to dietary excess<br>References:<br>ALK7 expression is specific for adipose tissue, reduced in obesity and correlates to factors implicated in metabolic disease [22]<br><br>Acute inhibition of ALK7 in adult mice by a chemical-genetic approach reduced diet-induced weight gain, fat accumulation, and adipocyte size, and enhanced adipocyte lipolysis and β-adrenergic signaling [23]

	Receptor	Evidence for metabolic roles in adipose and other tissues
		Mutant mice lacking ALK7 showed reduced fat accumulation and partial resistance to diet-induced obesity. They suggest that ALK7 receptor regulates adipose-tissue homeostasis and energy balance under nutrient overload [24]
2	APCDD1	Downregulation of APCDD1 in diet induced obesity impairs adipocyte differentiation and causes obesity related metabolic diseases [25]<br><br>Ligand:<br>GO biological process: negative regulation of fibroblast growth factor receptor signaling pathway, positive regulation of endocytosis<br><br>Wnt5a regulate insulin secretion [26]<br>Wnt5a regulate obesity [27], and role in metabolic inflammation [28]
3	CALCRL	GO process: cellular response to sucrose stimulus
4	CD36	GO molecular functions: high-density lipoprotein particle binding, lipoteichoic acid immune receptor activity, low-density lipoprotein particle binding, low-density lipoprotein particle receptor activity<br>GO biological processes: cellular response to lipoteichoic acid, cholesterol import, energy homeostasis, intestinal cholesterol absorption, lipid storage, lipoprotein transport, long-chain fatty acid import into cell, low-density lipoprotein particle clearance, low-density lipoprotein particle mediated signaling, positive regulation of cholesterol storage, sensory perception of taste, triglyceride transport<br><br>Implicated in metabolic complications of obesity [29]
5	CD81 (CD81 antigen)	GO molecular function: cholesterol binding<br>GO biological process: cellular response to low-density lipoprotein particle stimulus
6	CNTFR (Ciliary neurotrophic factor receptor subunit alpha)	Ligand CNTF:<br>GO - Molecular function: growth factor activity<br><br>Its ligand, CNTF, regulates adiposity and metabolic remodeling in adipocytes promotes ciliary neurotrophic factor-mediated fat loss in obesity [30, 31]
7	CSF2RA – (Granulocyte-macrophage colony-stimulating factor receptor subunit alpha)	Involved in glucose and lipid biosynthesis [32]<br>Ligand: Granulocyte Macrophage-Colony Stimulating Factor (GM-CSF) a proinflammatory cytokine that has a central action to reduce food intake and body weight [33]
8	CSPG4/NG2 (Chondroitin sulfate proteoglycan 4)	GO biological process: platelet-derived growth factor receptor signaling pathway, positive regulation of peptidyl-tyrosine phosphorylation, tissue remodeling, cell population proliferation, chondroitin sulfate biosynthetic process, chondroitin sulfate catabolic process, dermatan sulfate biosynthetic process<br>Glucose and Insulin tolerance is Impaired in NG2 null mice [34]
9	DDR1 (Epithelial discoidin domain-containing receptor 1)	GO - Biological process: regulation of cell growth, cell adhesion, multicellular organism development, regulation of cell-matrix adhesion, regulation of extracellular matrix disassembly, collagen binding<br><br>DDR1 deletion results in decreased obesity, and increased energy expenditure and brown fat activity, DDR1 expression was increased in adipose and correlated with obesity and fibrosis [35]
10	EDNRB (Endothelin receptor type B) maybe yes or no. Seesm more in visceral	GO biological process: cellular response to lipopolysaccharide, positive regulation of cell population proliferation<br><br>Insulin induce EDNRB expression [36]
11	F3 (tissue factor)	GO molecular function: phospholipid binding

	Receptor	Evidence for metabolic roles in adipose and other tissues
		GO biological process: positive regulation of cell migration, positive regulation of endothelial cell proliferation, positive regulation of platelet-derived growth factor receptor signaling pathway<br><br>F3 increased the activity and mRNA levels of insulin receptor, and the latter also increased the mRNA levels of IRS-1. F3 increased the protein levels of Insulin receptor [37]
12	FZD4 (Frizzled-4)	GO biological process: regulation of vascular endothelial growth factor receptor signaling pathway, multicellular organism development, negative regulation of cell-substrate adhesion<br><br>Is important in glucose and energy metabolism [38]<br>Insulin activates wnt signaling than binds the frizzle receptors (FZD4, FZD5) [39]<br>Hyperinsulinemia resulted in a decrease in adipose tissue FZD4 [40]<br>Fzd4 and fzd5 expression was downregulated in the islets of mice fed the HF/HS diet [41]<br>The activation of Frizzled receptor and Wnt signaling in pancreatic islets resulted in higher insulin secretion and an increase in beta cell proliferation, thus leading to islet adaptation in a pre-diabetic state [42]<br>Secreted Wnt6 mediates diabetes-associated centrosome amplification via its receptor FZD4 [43]
13	FZD5 (Frizzled-5)	GO molecular function: lipid binding<br>GO biological process: negative regulation of cell population proliferation, regulation of bicellular tight junction assembly<br><br>Related to insulin transcription and secretion in beta cells [44]
14	GIPR (Gastric inhibitory polypeptide receptor)	GO molecular function: gastric inhibitory peptide receptor activity<br>GO biological process: endocrine pancreas development, generation of precursor metabolites and energy, positive regulation of insulin secretion, regulation of insulin secretion, response to fatty acid, response to glucose, response to nutrient
15	GLP2R (Glucagon-like peptide-2 Receptor)	GO molecular function: glucagon receptor activity<br>Regulates energy balance and glucose homeostasis [45]<br>Enhance hepatic insulin sensitivity [46]
16	GPIHBP1 (Glycosylphosphatidylinositol-anchored high density lipoprotein-binding protein 1)	GO molecular function: lipid binding, lipoprotein particle binding<br>GO biological process: cholesterol homeostasis, positive regulation of lipoprotein lipase activity, regulation of lipoprotein lipase activity, retinoid metabolic process, triglyceride homeostasis
17	KLB (Beta-klotho)	GO molecular function: fibroblast growth factor binding, fibroblast growth factor receptor binding, hydrolase activity, hydrolyzing O-glycosyl compounds<br>GO biological process: carbohydrate metabolic process, fibroblast growth factor receptor signaling pathway, positive regulation of cell population proliferation, positive regulation of MAPKKK cascade by fibroblast growth factor receptor signaling pathway
18	LRP5 (Low-density lipoprotein receptor-related protein 5)	GO biological process: adipose tissue development, canonical Wnt signaling pathway, cholesterol homeostasis, cholesterol metabolic process, endocytosis, glucose catabolic process, positive regulation of cell population proliferation, positive regulation of fat cell differentiation, positive regulation of mesenchymal cell proliferation, regulation of insulin secretion involved in cellular response to glucose stimulus<br>Low-density lipoprotein receptor-related protein 5 (LRP5) is essential for normal cholesterol metabolism and glucose-induced insulin secretion [47]<br>Regulates human body fat, contributes to lipoprotein metabolism [48]<br>LRP5 modifies energy metabolism by positively affecting insulin signaling. Altered Wnt/LRP5 activity can play a role in obesity and insulin resistance [49]<br>LRP5 promotes adipocyte insulin sensitivity [50]

	Receptor	Evidence for metabolic roles in adipose and other tissues
19	MET (Hepatocyte growth factor receptor)	GO biological process: endothelial cell morphogenesis, liver development, multicellular organism development<br><br>Met and Insulin receptor hybrid governs hepatic glucose metabolism [51]<br>The ligand, (HGF), is an important component of the pathophysiology of insulin receptor, insulin resistance and diabetes [52]
20	NMUR1 (Neuromedin-U receptor 1)	Neuromedin U suppresses glucose-stimulated insulin secretion in pancreatic β cells [53]<br>The discovery of neuromedin U and its pivotal role in the central regulation of energy homeostasis [54]
21	NOTCH4 (Neurogenic locus notch homolog protein 4)	Notch signaling is a regulator of metabolism [55]
22	NPR1 (Atrial natriuretic peptide receptor)	GO biological process: negative regulation of cell growth, negative regulation of smooth muscle cell proliferation, dopamine metabolic process<br><br>Is regulated by insulin in adipose cells [56]
23	NPR3 (Atrial natriuretic peptide receptor3)	GO biological process: dopamine metabolic process, negative regulation of cell growth<br><br>Reciprocal regulation of natriuretic peptide receptors by insulin in adipose cells [56]<br>Adipose tissue natriuretic peptide receptor expression is related to insulin sensitivity in obesity and diabetes [57]
24	OPRL1 (Nociceptin receptor) – opioid receptor	GO biological process: eating behavior, positive regulation of gastric acid secretion<br><br>Regulate diet preferences[58]<br>The ligand regulates energy homeostasis [59]
25	P2RY14/GPR105 P2Y purinoceptor 14	GPR105 ablation prevents inflammation and improves insulin sensitivity in mice with diet-induced obesity [60]
26	PGRMC1 (Progesterone Receptor Membrane Component 1) Novel cell surface receptor	Increases plasma levels of insulin [61]<br>PGRMC1 modulates insulin receptor signaling and function in adipose cells. Adipose-specific PGRMC2-null mice unable to activate adaptive thermogenesis and prone to greater metabolic deterioration when fed a high-fat diet. By contrast, obese-diabetic mice treated with a small-molecule PGRMC2 activator showed substantial improvement of diabetic features. Modulation of PGRMC2 is suggested to revert obesity-linked defects in adipocytes [62]<br>Involved in glucose metabolism and energy metabolism: [63] mutational manipulation of PGRMC1 phosphorylation status in MIA PaCa-2 (MP) pancreatic cancer cells exhibit reduced levels of proteins involved in energy metabolism and mitochondrial function, and altered glucose metabolism<br>Involved in metabolism [64]<br>Involved in lipogenesis regulation. *Pgrmc1* suggested to be involves with the first step in regulating the hepatic de novo lipogenesis under an excess energy condition, tested on *Pgrmc1* knockout (KO) mice showed significant increases in hepatic accumulation [65]
27	PLXNA4 (Plexin-A4)	High glucose up-regulates the ligand Semaphorin 3A expression [66]<br>The ligand Semaphorin3a promotes advanced diabetic nephropathy [67]
28	PTPRF (Receptor-type tyrosine-protein phosphatase F)	PTPRF/LAR inhibit the insulin receptor activation [68]
29	SORT1 (Sortilin)	GO biological process: glucose import, response to insulin, negative regulation of fat cell differentiation, negative regulation of lipoprotein lipase activity, endocytosis<br><br>Sort1 loss-of-function was shown to decrease insulin-dependent glucose uptake into the adipocytes [69]

	Receptor	Evidence for metabolic roles in adipose and other tissues
30	TYRO3 (one of Tyro3, Axl and MerTK (TAM receptors), The TAMs and their ligands—Gas6 and Protein S). tumor-associated macrophages (TAMs)	GO biological process: substrate adhesion-dependent cell spreading<br><br>The ligand GAS6 and its receptors (e.g., TYRO3) are involved in the pathogenesis of obesity and insulin resistance [70]
31	VIPR1/ VPAC1 (Vasoactive intestinal polypeptide receptor 1)	GO biological process: positive regulation of cell population proliferation<br><br>Activation of VPAC1 has been implicated in elevating glucose output [71]
32	NPY5R	NPY5R is an important mediator of energy homeostasis in rodents [72]<br>Regulates energy homeostasis [73]<br>Related to obesity [74]<br>Suggested as a drug target for anti-obesity [75]
33	ERAP1	Suggested as a treatment to T1diabetes [76]<br>preprint:<br>ERAP1 hepatokine impairs SM (skeletal muscle) and whole-body insulin sensitivity via ADRB2/PKA pathway, and its inhibition might provide a therapeutic strategy for diabetes, particularly for those with SM insulin resistance [77]
34	ADORA1 (Adenosine receptor 1) The activity of this receptor is mediated by G proteins which inhibit adenylyl cyclase	GO biological process: fatty acid homeostasis, lipid catabolic process, triglyceride homeostasis<br><br>ADORA1 regulate metabolism and islet endocrine and vascular functions during ageing, including via the modulation of oxidative stress and inflammatory responses [78]<br>ADORA1R signaling contributes importantly to insulin-controlled glucose homeostasis and insulin sensitivity in C57BL/6 mice and is involved in the metabolic regulation of adipose tissue [79]
35	S1PR4	GO molecular function: lipid binding<br>GO biological process: regulation of metabolic process

Table S3. Enrichment results of 55 negatively labeled receptors (negative rate > 0.8) inferred by PU SVM bagging algorithm.

Term	Overlap	P-value	Adjusted P-value	Odds Ratio	Combined Score	Genes
**Cytokine-cytokine receptor interaction**	17/294	1.81E-18	5.59E-16	21.0265925	858.94827 9	NGFR;TNFRSF6B;CSF3R;IL4R;TNFRSF10B;CSF2RB;TNFRSF11B;TNFRSF10A;OSMR;TNFRSF1B;TNFRSF1A;IL3RA;ACKR4;ACKR3;CCR7;TNFRSF14;IL12RB1
Pathways in cancer	11/530	1.70E-07	1.75E-05	7.54716981	117.64159 6	RET;PDGFRA;CSF3R;IL4R;IL3RA;FZD6;BDKRB2;BDKRB1;TRAF2;CSF2RB;IL12RB1
Apoptosis	7/143	1.26E-07	1.93E-05	17.8003814	282.85772	NGFR;IL3RA;TNFRSF10B;TRAF2;CSF2RB;TNFRSF10A;TNFRSF1A

**Neuroactive ligand-receptor interaction**	9/338	3.24E-07	2.00E-05	9.68262507	144.671437	P2RX7;PTGIR;HRH1;HRH2;BDKRB2;APLNR;FPR1;BDKRB1;TACR1
JAK-STAT signaling pathway	7/162	2.94E-07	2.26E-05	15.7126824	236.31167 5	PDGFRA;CSF3R;IL4R;IL3RA;CSF2RB;OSMR;IL12RB1
Calcium signaling pathway	7/188	8.04E-07	4.13E-05	13.5396518	190.01540 9	P2RX7;PDGFRA;HRH1;HRH2;BDKRB2;BDKRB1;TACR1
PI3K-Akt signaling pathway	8/354	5.25E-06	2.31E-04	8.21777093	99.909410 4	PDGFRA;NGFR;CSF3R;IL4R;ITGB3;IL3RA;OSMR;TLR2
Hematopoietic cell lineage	May-97	6.94E-06	2.67E-04	18.7441425	222.63821	CSF3R;IL4R;ITGB3;IL3RA;CD44
Osteoclast differentiation	5/127	2.57E-05	8.81E-04	14.3163923	151.29237 8	ITGB3;TRAF2;TNFRSF11B;SIRPB1;TNFRSF1A
Cell adhesion molecules (CAMs)	5/145	4.86E-05	0.00149598	12.539185	124.54534 5	CNTNAP1;SELL;NRXN2;F11R;SELE

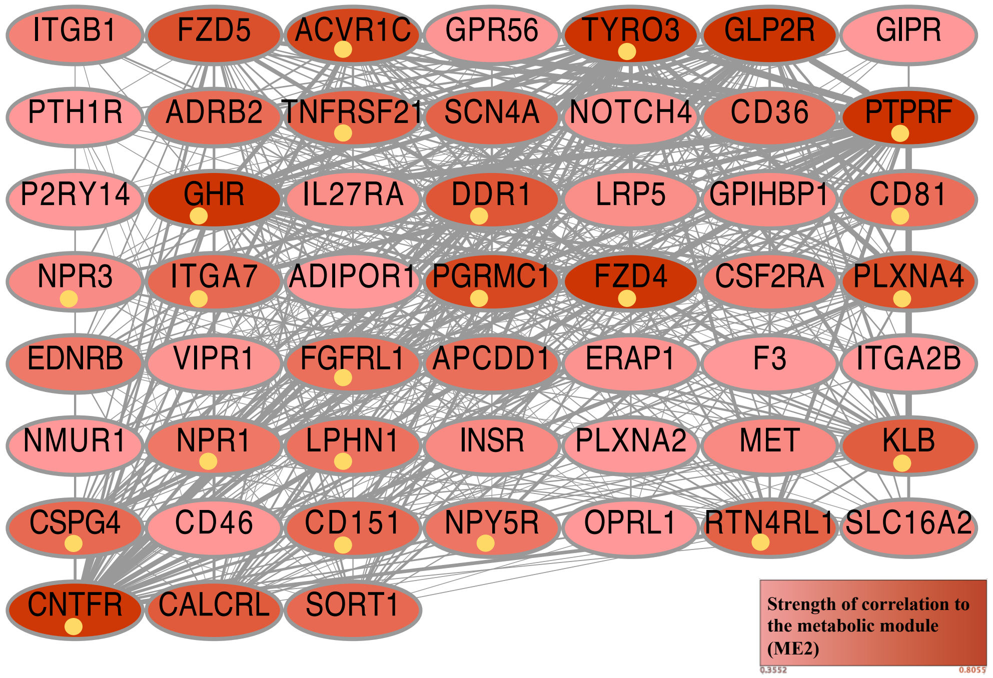

Figure S1. Predicted metabolic receptors in adipose and similar tissues. a. Key driver receptors of the Adipose–Subcutaneous metabolic module (ME1). The strength of the colour represents the module's receptor key drivers, i.e., the strength of the correlation between the receptor and the eigengene of the metabolic module (i.e., the first principal component of the module). The module eigengene corresponds to the first principal component of a given module and is considered the most representative gene expression in a module. It can be seen that among others, the known growth hormone receptor (GHR) and insulin receptor (INSR) are predicted to have a metabolic function and are correlated with the metabolic module (r^2=0.8 and r^2=0.42, respectively). The edge width represents the weight of the receptor co-expression values generated by the WGCNA algorithm, based on correlation values and topological similarity (TOM, adjacent nodes that have similar neighbours) between the receptors. Receptors that are also predicted to have a metabolic function in Adipose–Visceral are highlighted with yellow circles.

References

1. Antonescu, C. N., McGraw, T. E. & Klip, A. Reciprocal regulation of endocytosis and metabolism. *Cold Spring Harb. Perspect. Biol.* **6**, (2014).
2. Vijayakumar, A., Yakar, S. & LeRoith, D. The intricate role of growth hormone in metabolism. *Frontiers in Endocrinology* vol. 2 32 (2011).
3. Luo, L. & Liu, M. Adipose tissue in control of metabolism. *Journal of Endocrinology* vol. 231 R77–R99 (2016).
4. Cignarelli, A. *et al.* Insulin and insulin receptors in adipose tissue development. *International Journal of Molecular Sciences* vol. 20 (2019).
5. Zhang, J. & Liu, F. Tissue-specific insulin signaling in the regulation of metabolism and aging. *IUBMB Life* vol. 66 485–495 (2014).
6. Rask-Madsen, C. & Kahn, C. R. Tissue-specific insulin signaling, metabolic syndrome, and cardiovascular disease. *Arterioscler. Thromb. Vasc. Biol.* **32**, 2052–2059 (2012).
7. Sun, L. Y. & Bartke, A. Tissue-Specific GHR Knockout Mice: Metabolic Phenotypes. *Front. Endocrinol. (Lausanne).* **5**, 243 (2015).
8. Vijayakumar, A., Yakar, S. & LeRoith, D. The intricate role of growth hormone in metabolism. *Frontiers in Endocrinology* vol. 2 32 (2011).
9. Bjursell, M. *et al.* Opposing effects of adiponectin receptors 1 and 2 on energy metabolism. *Diabetes* **56**, 583–593 (2007).
10. Blüher, M. *et al.* Gene expression of adiponectin receptors in human visceral and subcutaneous adipose tissue is related to insulin resistance and metabolic parameters and is altered in response to physical training. *Diabetes Care* **30**, 3110–3115 (2007).
11. Pereira, S. *et al.* Metabolic effects of leptin receptor knockdown or reconstitution in adipose tissues. *Sci. Rep.* **9**, 1–17 (2019).
12. Gao, Y. *et al.* Adipocyte iron regulates leptin and food intake. *J. Clin. Invest.* **125**, 3681–3691 (2015).
13. Gabrielsen, J. S. *et al.* Adipocyte iron regulates adiponectin and insulin sensitivity. *J. Clin. Invest.* **122**, 3529–3540 (2012).
14. McClain, D. A. *et al.* Adipose tissue transferrin and insulin resistance. *J. Clin. Endocrinol. Metab.* **103**, 4197–4208 (2018).
15. Gotardo, É. M. F. *et al.* Mice that are fed a high-fat diet display increased hepcidin expression in adipose tissue. *J. Nutr. Sci. Vitaminol. (Tokyo).* **59**, 454–461 (2013).
16. Bordicchia, M. *et al.* PCSK9 is expressed in human visceral adipose tissue and regulated by insulin and cardiac natriuretic peptides. *Int. J. Mol. Sci.* **20**, (2019).
17. Borcherding, D. C. *et al.* Dopamine receptors in human adipocytes: Expression and functions. *PLoS One* **6**, (2011).
18. Caron, A. *et al.* Adipocyte Gs but not Gi signaling regulates whole-body glucose homeostasis. *Mol. Metab.* **27**, 11–21 (2019).
19. Garg, A., Sankella, S., Xing, C. & Agarwal, A. K. Whole-exome sequencing identifies ADRA2A mutation in atypical familial partial lipodystrophy. *JCI Insight* **1**, (2016).
20. Zhang, H. *et al.* Cardiovascular and metabolic phenotypes in relation to the ADRA2B insertion/deletion polymorphism in a Chinese population. *J. Hypertens.* **23**, 2201–2207 (2005).
21. Ceasrine, A. M., Lin, E. E., Lumelsky, D. N., Iyer, R. & Kuruvilla, R. Adrb2 controls glucose homeostasis by developmental regulation of pancreatic islet vasculature. *Elife* **7**, (2018).
22. Carlsson, L. M. S. *et al.* ALK7 expression is specific for adipose tissue, reduced in

obesity and correlates to factors implicated in metabolic disease. *Biochem. Biophys. Res. Commun.* **382**, 309–314 (2009).
23. Guo, T. *et al.* Adipocyte ALK7 links nutrient overload to catecholamine resistance in obesity. *Elife* **3**, e03245 (2014).
24. Andersson, O., Korach-Andre, M., Reissmann, E., Ibáñez, C. F. & Bertolino, P. Growth/differentiation factor 3 signals through ALK7 and regulates accumulation of adipose tissue and diet-induced obesity. *Proc. Natl. Acad. Sci. U. S. A.* **105**, 7252–7256 (2008).
25. Yiew, N. K. H. *et al.* A novel role for the Wnt inhibitor APCDD1 in adipocyte differentiation: Implications for diet-induced obesity. *J. Biol. Chem.* **292**, 6312–6324 (2017).
26. Xu, W. *et al.* Islet Stellate Cells Regulate Insulin Secretion via Wnt5a in Min6 Cells. *Int. J. Endocrinol.* **2020**, (2020).
27. Fuster, J. J. *et al.* Noncanonical wnt signaling promotes obesity-induced adipose tissue inflammation and metabolic dysfunction independent of adipose tissue expansion. *Diabetes* **64**, 1235–1248 (2015).
28. Relling, I. *et al.* Role of wnt5a in Metabolic Inflammation in Humans. *J. Clin. Endocrinol. Metab.* **103**, 4253–4264 (2018).
29. Love-Gregory, L. & Abumrad, N. A. CD36 genetics and the metabolic complications of obesity. *Current Opinion in Clinical Nutrition and Metabolic Care* vol. 14 527–534 (2011).
30. Pasquin, S., Sharma, M. & Gauchat, J. F. Ciliary neurotrophic factor (CNTF): New facets of an old molecule for treating neurodegenerative and metabolic syndrome pathologies. *Cytokine and Growth Factor Reviews* vol. 26 507–515 (2015).
31. Crowe, S., Turpin, S. M., Ke, F., Kemp, B. E. & Watt, M. J. Metabolic remodeling in adipocytes promotes ciliary neurotrophic factor-mediated fat loss in obesity. *Endocrinology* **149**, 2546–2556 (2008).
32. Naruse, K. *et al.* Involvement of Visceral Adipose Tissue in Immunological Modulation of Inflammatory Cascade in Preeclampsia. *Mediators Inflamm.* **2015**, (2015).
33. Kim, D. H. *et al.* The role of GM-CSF in adipose tissue inflammation. *Am. J. Physiol. - Endocrinol. Metab.* **295**, (2008).
34. Chang, Y. *et al.* Ablation of ng2 proteoglycan leads to deficits in brown fat function and to adult onset obesity. *PLoS One* **7**, e30637 (2012).
35. Lino, M. *et al.* Discoidin domain receptor 1-deletion ameliorates fibrosis and promotes adipose tissue beiging, brown fat activity, and increased metabolic rate in a mouse model of cardiometabolic disease. *Mol. Metab.* **39**, 101006 (2020).
36. Park, K. *et al.* Insulin decreases atherosclerosis by inducing endothelin receptor B expression. *JCI Insight* **1**, (2016).
37. Xiao, C., Wu, Q., Xie, Y., Zhang, J. & Tan, J. Hypoglycemic effects of Grifola frondosa (Maitake) polysaccharides F2 and F3 through improvement of insulin resistance in diabetic rats. *Food Funct.* **6**, 3567–3575 (2015).
38. Elghazi, L. *et al.* Importance of β-catenin in glucose and energy homeostasis. *Sci. Rep.* **2**, (2012).
39. Kim, M.-H., Hong, S.-H. & Lee, M.-K. Insulin Receptor-Overexpressing β-Cells Ameliorate Hyperglycemia in Diabetic Rats through Wnt Signaling Activation. *PLoS One* **8**, e67802 (2013).
40. Karczewska-Kupczewska, M., Stefanowicz, M., Matulewicz, N., Nikołajuk, A. & Straczkowski, M. Wnt signaling genes in adipose tissue and skeletal muscle of humans with different degrees of insulin sensitivity. *J. Clin. Endocrinol. Metab.* **101**, 3079–

3087 (2016).
41. Kurita, Y. *et al.* A high-fat/high-sucrose diet induces WNT4 expression in mouse pancreatic β-cells. *Kurume Med. J.* **65**, 55–62 (2018).
42. Kozinski, K. *et al.* Adipose- and muscle-derived Wnts trigger pancreatic β-cell adaptation to systemic insulin resistance. *Sci. Rep.* **6**, 1–12 (2016).
43. He, Q. J. *et al.* Secreted Wnt6 mediates diabetes-associated centrosome amplification via its receptor FZD4. *Am. J. Physiol. - Cell Physiol.* **318**, C48–C62 (2020).
44. Zhang, F. *et al.* Obesity-induced overexpression of miR-802 impairs insulin transcription and secretion. *Nat. Commun.* **11**, 1–16 (2020).
45. Guan, X. The CNS glucagon-like peptide-2 receptor in the control of energy balance and glucose homeostasis. *Am. J. Physiol. - Regul. Integr. Comp. Physiol.* **307**, R585–R596 (2014).
46. Shi, X. *et al.* Central GLP-2 enhances hepatic insulin sensitivity via activating PI3K signaling in POMC neurons. *Cell Metab.* **18**, 86–98 (2013).
47. Fujino, T. *et al.* Low-density lipoprotein receptor-related protein 5 (LRP5) is essential for normal cholesterol metabolism and glucose-induced insulin secretion. *Proc. Natl. Acad. Sci. U. S. A.* **100**, 229–234 (2003).
48. Loh, N. Y. *et al.* LRP5 regulates human body fat distribution by modulating adipose progenitor biology in a dose- and depot-specific fashion. *Cell Metab.* **21**, 262–273 (2015).
49. Palsgaard, J. *et al.* Cross-talk between insulin and Wnt signaling in preadipocytes: Role of Wnt co-receptor low density lipoprotein receptor-related protein-5 (LRP5). *J. Biol. Chem.* **287**, 12016–12026 (2012).
50. Loh, N. Y. *et al.* LRP5 promotes adipose progenitor cell fitness and adipocyte insulin sensitivity Short title: LRP5 and adipose tissue biology. *bioRxiv* 2020.03.04.976647 (2020) doi:10.1101/2020.03.04.976647.
51. Fafalios, A. *et al.* A hepatocyte growth factor receptor (Met)-insulin receptor hybrid governs hepatic glucose metabolism. *Nat. Med.* **17**, 1577–1584 (2011).
52. Oliveira, A. G. *et al.* The role of Hepatocyte Growth Factor (HGF) in insulin resistance and diabetes. *Frontiers in Endocrinology* vol. 9 503 (2018).
53. Zhang, W. *et al.* Neuromedin U suppresses glucose-stimulated insulin secretion in pancreatic β cells. *Biochem. Biophys. Res. Commun.* **493**, 677–683 (2017).
54. Kirsz, K. & Zięba, D. A. Odkrycie neuromedyny U i jej rola w centralnej regulacji homeostazy energetycznej. *Postepy Higieny i Medycyny Doswiadczalnej* vol. 66 196–203 (2012).
55. Bi, P. & Kuang, S. Notch signaling as a novel regulator of metabolism. *Trends in Endocrinology and Metabolism* vol. 26 248–255 (2015).
56. Nakatsuji, H. *et al.* Reciprocal regulation of natriuretic peptide receptors by insulin in adipose cells. *Biochem. Biophys. Res. Commun.* **392**, 100–105 (2010).
57. Kovacova, Z. *et al.* Adipose tissue natriuretic peptide receptor expression is related to insulin sensitivity in obesity and diabetes. *Obesity* **24**, 820–828 (2016).
58. Koizumi, M., Cagniard, B. & Murphy, N. P. Endogenous nociceptin modulates diet preference independent of motivation and reward. *Physiol. Behav.* **97**, 1–13 (2009).
59. Hernandez, J. *et al.* Nociceptin/orphanin FQ modulates energy homeostasis through inhibition of neurotransmission at VMN SF-1/ARC POMC synapses in a sex- and diet-dependent manner. *Biol. Sex Differ.* **10**, 1–24 (2019).
60. Xu, J. *et al.* GPR105 Ablation Prevents Inflammation and Improves Insulin Sensitivity in Mice with Diet-Induced Obesity. *J. Immunol.* **189**, 1992–1999 (2012).
61. Hampton, K. K., Anderson, K., Frazier, H., Thibault, O. & Craven, R. J. Insulin receptor plasma membrane levels increased by the progesterone receptor membrane

component 1. *Mol. Pharmacol.* **94**, 665–673 (2018).
62. Galmozzi, A. *et al.* PGRMC2 is an intracellular haem chaperone critical for adipocyte function. *Nature* **576**, 138–142 (2019).
63. Thejer, B. M. *et al.* PGRMC1 phosphorylation affects cell shape, motility, glycolysis, mitochondrial form and function, and tumor growth. *BMC Mol. Cell Biol.* **21**, (2020).
64. Thejer, B. M. *et al.* PGRMC1 effects on metabolism, genomic mutation and CpG methylation imply crucial roles in animal biology and disease. *BMC Mol. Cell Biol.* **21**, 26 (2020).
65. Lee, S. R. *et al.* Loss of progesterone receptor membrane component 1 promotes hepatic steatosis via the induced de novo lipogenesis. *Sci. Rep.* **8**, (2018).
66. Wu, L. yan *et al.* High glucose up-regulates Semaphorin 3A expression via the mTOR signaling pathway in keratinocytes: A potential mechanism and therapeutic target for diabetic small fiber neuropathy. *Mol. Cell. Endocrinol.* **472**, 107–116 (2018).
67. Aggarwal, P. K. *et al.* Semaphorin3a promotes advanced diabetic nephropathy. *Diabetes* **64**, 1743–1759 (2015).
68. De Lorenzo, C., Greco, A., Fiorentino, T. V., Mannino, G. C. & Hribal, M. L. Variants of insulin-signaling inhibitor genes in type 2 diabetes and related metabolic abnormalities. *International Journal of Genomics* vol. 2013 (2013).
69. Li, J. *et al.* Inhibition of insulin/PI3K/AKT signaling decreases adipose Sortilin 1 in mice and 3 T3-L1 adipocytes. *Biochim. Biophys. Acta - Mol. Basis Dis.* **1863**, 2924–2933 (2017).
70. Hsiao, F.-C. *et al.* Circulating Growth Arrest-Specific 6 Protein Is Associated With Adiposity, Systemic Inflammation, and Insulin Resistance Among Overweight and Obese Adolescents. *J. Clin. Endocrinol. Metab.* **98**, E267–E274 (2013).
71. Tsutsumi, M. *et al.* A potent and highly selective VPAC2 agonist enhances glucose-induced insulin release and glucose disposal: A potential therapy for type 2 diabetes. *Diabetes* **51**, 1453–1460 (2002).
72. Mashiko, S. *et al.* Effects of a novel Y5 antagonist in obese mice: Combination with food restriction or sibutramine. *Obesity* **16**, 1510–1515 (2008).
73. Mashiko, S. *et al.* Synergistic interaction between neuropeptide Y1 and Y5 receptor pathways in regulation of energy homeostasis. *Eur. J. Pharmacol.* **615**, 113–117 (2009).
74. Moriya, R. *et al.* Comparison of independent and combined chronic anti-obese effects of NPY Y2 receptor agonist, PYY(3-36), and NPY Y5 receptor antagonist in diet-induced obese mice. *Peptides* **30**, 1318–1322 (2009).
75. Levens, N. R. & Della-Zuana, O. Neuropeptide Y Y5 receptor antagonists as anti-obesity drugs. *Current Opinion in Investigational Drugs* vol. 4 1198–1204 (2003).
76. Thomaidou, S. *et al.* β-cell stress shapes CTL immune recognition of preproinsulin signal peptide by posttranscriptional regulation of endoplasmic reticulum aminopeptidase 1. *Diabetes* **69**, 670–680 (2020).
77. Guo, F. *et al.* Hepatokine ERAP1 impairs skeletal muscle insulin sensitivity via ADRB2/PKA pathway. (2020) doi:10.21203/rs.3.rs-37586/v1.
78. Yang, T. *et al.* Abrogation of adenosine A1 receptor signalling improves metabolic regulation in mice by modulating oxidative stress and inflammatory responses. *Diabetologia* **58**, 1610–1620 (2015).
79. Faulhaber-Walter, R. *et al.* Impaired glucose tolerance in the absence of adenosine A1 receptor signaling. *Diabetes* **60**, 2578–2587 (2011).
